# The Implementation of Decision Aids During Medical Consultations for Lung Cancer Patients: A Focus Group Within I3LUNG Project

**DOI:** 10.1007/s13187-025-02566-6

**Published:** 2025-01-21

**Authors:** Valeria Sebri, Patrizia Dorangricchia, Dario Monzani, Chiara Marzorati, Roberto Grasso, Lorenzo Conti, Giuseppe Lo Russo, Leonardo Provenzano, Andra Diana Dumitrascu, Gabriella Pravettoni

**Affiliations:** 1 Applied Research Division for Cognitive and Psychological Science, IEO, European Institute of Oncology IRCCS, Via Giuseppe Ripamonti, 435, 20141 Milan, Italy; 2 Laboratory of Behavioral Observation and Research on Human Development, Department of Psychology, Educational Science and Human Movement, University of Palermo, 90128 Palermo, Italy; 3 Department of Oncology and Hemato-Oncology, University of Milan, Milan, Italy; 4 Thoracic Oncology Unit, Medical Oncology Department 1, Fondazione IRCCS Istituto Nazionale Tumori, Milan, Italy; 5 Medical Oncology Department, Fondazione IRCCS Istituto Nazionale dei Tumori di Milano, Milan, Italy

**Keywords:** Lung cancer patients, Decision aids, Shared decision-making, Doctor-patient relationship, Focus group

## Abstract

Lung cancer patients generally receive several information regarding their illness characteristics and available intervention. Therefore, patients can experience confusion, leading to anxiety and distress that might damage the relationship with physicians and treatment adherence. Literature showed that implementing decision aid tools during consultation can promote patients’ knowledge and awareness about lung cancer and available oncological intervention, improving a shared decision-making process. However, not all lung cancer patients always appreciate decision aids’ implementation. The present qualitative study explored patients’ opinions and preferences regarding the implementation of decision aids during medical consultation. Twenty-two lung cancer patients who have already attended medical consultations for lung cancer voluntarily participated in four online focus groups carried out between January 2023 and December 2024. A thematic analysis with a bottom-up approach highlighted three main themes: the typology and number of information that patients would have received, the relevance of patient-and-doctor relationship, and the effects of providing additional decision-making tools on patients’ emotions and preferences. Findings showed controversial opinions among patients, highlighting the relevance of personalized intervention tailored to patients’ preferences. Practical implications are given.

## Introduction

Lung cancer is the most common cause of cancer-related death in Italy and worldwide. According to the World Health Organization, lung cancer incidence and mortality rates are highest in individuals between 65 and 84 years old and in developed countries [1]. Literature showed two main types of lung cancer: non-small cell lung cancer (NSCLC) and small cell lung cancer (SCLC). NSCLC is the most common type of lung cancer, accounting for 85% of patients. Frequent symptoms include cough, dyspnea, anorexia, hemoptysis, fatigue, and chest pain, which impair Quality of Life (QoL) strongly [2, 3]. In general, there are different oncological treatment options for patients with NSCLC, according to their disease stage and characteristics,moreover, more than one oncological treatment in combination or consecutively with each other must be often implemented [4]. Physicians use to present lung cancer patients with all possible treatment options during oncological consultations. Interestingly, studies evidenced that patients reported high levels of unmet supportive care needs and many doubts about treatment options and ruminative thinking, which often remain unresolved after medical consultations [5, 6]. More specifically, patients would like to receive more information about lung cancer symptoms and available treatments, with a specific interest in their related benefits and harms [7]. In a study by Li and Girgis [8], patients strongly appreciated knowing lung cancer characteristics and having indications about how they would have to cope with the physical effects of the disease and treatment demands, promoting the management of psychological distress. In line with this, a systematic-review study by Cochrane and colleagues [9] identified a broad range of needs associated with the lung cancer pathway. The authors stated that some needs and emotions are more pervasive in lung cancer patients compared to other types of cancer. For example, lung cancer patients experience high anxiety and depression scores [10]; at the same time, they are at greater risk of suicide compared to other cancer patients due to poor prognosis, the side-effects of aggressive treatments, and physical limitations that generate severe frustration [11]. Moreover, persisting physical issues lead to long-term effects on their daily functioning (e.g., ability in the returning to work, walk, and self-care) because of a lack of energy and severe pain [12]. To sum up, symptom burden and psychological distress are closely linked to lung cancer patients’ unmet needs and low awareness about their illness and their scarce tendency to cope with the disease [13, 14].

Despite this background, lung cancer patients’ psychological needs are not often addressed and managed by the health care system. Current research highlights the relevance of psychological interventions to improve patients’ knowledge and their awareness about treatment preferences, enhancing compliance with physicians and physical and emotional well-being (i.e., psychological distress, anxiety, and depression scores decreased) [15, 16]. Monzani et al. [17] underlined that decisions about lung cancer treatments are preference-sensitive choices. Starting from available treatment options, patients might often be asked to choose between a therapeutic option with possible harmful effects on their QoL or an alternative treatment with less effectiveness but fewer damaging effects. Understanding patients’ preferences is essential to know patients’ willingness to tolerate risky outcomes. Thus, patient preferences may improve the decision-making process and their experience with the treatment, leading to better health outcomes [18]. Similarly, Leighl et al. [19] showed that patients engaged in shared decision-making showed improvements in their treatment options perception and positive emotions. Specifically, patients shared information and discussed treatment options to make the final decisions with their physician. Findings highlighted that lung cancer patients preferred to play an active role during health decisions by receiving accurate information about their illness and treatment choices. In other words, involving patients in their care contributed to better health outcomes, in terms of low anxiety, treatment adherence, and QoL. Additionally, recent studies [20] showed that shared decision-making helped patients to make informed decisions based on better illness knowledge, realistic expectations of outcomes, and personal values. To sum up, it is essential to develop tailored psychological interventions and health care services to assess and manage patients’ needs and preferences during their oncological journey. In this regard, current literature showed the effectiveness also of qualitative approaches, such as focus groups. Focus group is a qualitative method that identifies participants’ thoughts and emotions focused on the topic object of the investigation in a very short time [21]. In the oncological field, it can be a useful way to elicit public views on health and medicine, particularly from geographically dispersed participants [22, 23]. Moreover, it allows the examination of patients’ experiences and enables sensitive topics to be discussed, such as identifying the treatment characteristics and consequences, for example, different opinions about patients’ cancer disease and the treatment process in an online focus group conducted by Garcia and colleagues [24]. Similarly, Petrocchi and colleagues [25] highlighted the focus group’s efficacy in identifying patients’ preferred treatment, focusing on their trade-offs regarding treatment options. Moreover, a study by Looijmans et al. [26] reported the positive impact of participating in a focus group on the perception of peer social support, which is fundamental in the oncological field [27]. Authors reported that joining the focus group was experienced by participants as a supportive time to perceive social support and understanding from others.

Although supportive care needs of patients have been explored in the literature over the years, the relationship between lung cancer patients’ needs, preferences, QoL, and therapeutic adherence is still unclear [9]. To date, there is insufficient knowledge of patients’ information needs and, above all, how those information needs change over time. Patients reported unfulfilled needs in different areas of disease care; furthermore, healthcare providers did not always perceive patients’ needs correctly, leading to compromised efficacy of information provision [28]. The authors pointed out that patient-driven services would be helpful in identifying and addressing patients’ information needs and preferences. Starting from the present background, the main objective of the present focus group study will be to explore information needs and preferences about cancer treatments within the I3LUNG project. In line with the I3LUNG research goals, focus group information will be helpful in developing an ad hoc Individual Patient Decision Aid System (IPDAS) for lung cancer patients. IPDAS’ guidelines will sustain patients to clarify what are their options for treatments and preferences during medical consultation, thereby promoting their knowledge and their active participation in shared decision-making about their treatments.

## Methods

### Participants and Procedure

This study was performed in line with the principles of the Declaration of Helsinki and approved by the Institutional Review Board (IRB) of the National Tumor Institute, Milan (N. 147/22).

A total of 22 lung cancer patients were involved in four online focus groups within the I3LUNG project. Participants were all lung cancer patients who received one oncological treatment between chemotherapy, radiotherapy, and immunotherapy. They were aged 18 years or older and currently residing in Italy. Exclusion criteria were related to cognitive impairment, inability to understand the study or to sign the informed consent, and/or mental disorders that prohibited their participation in focus groups. Patients were recruited from Istituto Nazionale dei Tumori (Italy) where they received oncological treatments and interventions. Participants voluntarily agreed to participate in this study. In particular, they received an invitation email, and individuals interested in participating responded with their telephone number and a preferred time to call and discuss the study’s details. If they agreed to participate in the study, a follow-up email was sent, which contained an information sheet outlining the study, an informed consent form to sign, and sociodemographic questionnaires through the Qualtrics platform (e.g., age, sex, and educational level). Moreover, a ZOOM link to participate in the focus group intervention was sent. Focus groups were based on prompts designed to elicit the free expression of patients’ opinions about their experiences of medical consultations freely, in a narrative way. Conductors encouraged patients to share thoughts and emotions about their experiences without fear of judgment.

### Data Collection

Twenty-five emails were received from interested individuals; 22 attended the arranged time slots for one of the four focus groups. No compensation was offered for participation. People involved in the present study showed a mean age of 66.28 years old (age range = 44–72; SD = 8.67). Most of the patients were female (50%) and received a lung cancer diagnosis for less than 5 years (64%) (see more details in Table 1).

**Table 1:** Sociodemographic and clinical characteristics of participants (*n* = 22)

	Mean age, 66.28	SD, 8.67
	*N*	%
**Gender**		
Male	11	50%
Female	11	50%
**Years of education**		
5–10 years	10	45%
10–15 years	5	23%
15–20 years	7	32%
**How long have you been diagnosed?**		
< 5 years	14	64%
5–10 years	8	36%

The lead researcher (VS), a female researcher and psychologist at the European Institute of Oncology, facilitated the focus group in collaboration with two other psychologists of the same oncological institute (PD and RG). Before each focus group, the facilitator established a relationship with participants explaining the research goals and the structure of intervention. Moreover, the conductor shared that the focus group’s purpose was to explore individuals’ experiences regarding information received during medical consultations. The facilitator then prompted group discussion, using questions such as “What was your experience with the information you received when you were told about the treatment?” and “Was the information clear? Did you receive all the information you assessed important?” Participants were not given the list of questions prior to promote the focus group discussion [29]. All focus groups were audio-recorded, transcribed verbatim, and analyzed by the research team. Field notes were taken directly after each focus group discussion; then, authors (VS, PD, and LC) discussed thoughts, concerns, feelings, attitudes, beliefs, expectations, and potential biases to account for why certain elements were identified during data collection [30, 31].

### Focus Group Intervention

All lung cancer patients participated in only one focus group. To ensure that online discussion was manageable, focus groups were kept to between five and eight participants. Each group met virtually via a web video conferencing platform (Microsoft ZOOM) for between 90 and 120 min from January 2023 to December 2024. Participants joined using both video and audio. Starting from previous studies focused on lung cancer patients and their experiences with medical consultations [26], focus group contents were defined and scheduled by two researchers (CM and DM). Moreover, three expert psycho-oncologists with extensive professional experience in patient-and-doctor communication topics conducted the discussion in groups (VS, PD, and RG).

The topic guide for the focus groups was initially developed using existing literature on the implementation of decision aids (DAs) in lung cancer patients during medical consultations. The main topic was aimed at investigating the following: (1) patients’ preferences and comprehension in reference to their illness characteristics (i.e., “Do you think you have received and understood all the important information about the proposed treatment(s)?” and “When the doctor presented you with treatment options, were there any terms that you struggled to understand?”), (2) the involvement of other sources to collect information about treatment options (i.e., “Did you search for other information independently related to the treatment(s) or not? If yes, what information did you search for?”), (3) the best modality of information presentation (i.e., “In what modality would you have liked to receive information about the treatment(s)? For example, written text, video, brochure with concise but explanatory vignettes”), and (4) patients’ need of shared decision-making (i.e., “When the doctor presented the treatment options to you, were you interested in receiving as much information as possible about the treatment options or would you have preferred not to be involved in the choice?”). All lung cancer patients shared their illness experiences in the online group by exploring their personal opinions and related emotions and thoughts. Focus groups included a discussion between participants to share individuals’ points of view and listen to others’ experiences.

### Data Analysis

According to the study aims, data collection and analysis followed an iterative process, whereby emergent themes from early focus groups were used to add to or refine questions during subsequent focus groups. The analytical process followed the main phases of qualitative thematic analysis, as described by Braun and Clarke [32]. Adopting an inductive approach, we did not try to fit the data into a pre-existing frame, but, starting from initial prompts, sub-themes were identified through a pragmatic approach, whereby initial broad research questions inform the abductive generation of themes [33, 34]. Accordingly, a qualitative thematic analysis with a bottom-up approach was conducted, focusing the attention on the contents reported by participants [35]. In line with Braun and Clarke’s guidelines [35], we followed these steps to analyze data. Firstly, three authors (VS, PD, and LC) read each transcription many times to familiarize with and understand the focus groups’ contents. Moreover, themes were discussed and further developed with another researcher (RG) during virtual research group meetings. Second, an initial coding identified the main and more frequent segments of the textual reports. Therefore, the analytical approach of this study was mostly “semantic” [36]. Researchers considered a single segment when the answer reported a single main information regarding the patient-and-doctor consultation, and themes were identified within the explicit meanings of the data. In other words, we only looked for what participants shared. Then, initial primary (open) codes were developed and were then connected to other related themes to form overarching secondary codes that were developed into the three main themes described below [34, 37]. If one answer reported a single main or outcome, it was considered a single segment. The present coding procedure was run with Microsoft Excel. To help analysis, we looked to validate “sensitive moments” between groups that indicated complex but essential issues [38]. Negative case analysis was used to seek for information that did not fit emergent themes, and where this occurred, themes were modified accordingly [39]. Fourth, two authors (VS and PD) reviewed the thematic map, and possible discrepancies were solved by a fourth researcher (LC). Finally, researchers labeled the different codes, which were grouped into potential main themes and their related sub-themes to clarify explicit and defined contents. One author (DM), who did not take part in the previous phases of analysis, reviewed the coding process to further validate the thematic map.

## Results

An inductive thematic template analysis revealed three themes across all focus groups, specifically (i) typology and number of information, (ii) patient-and-doctor relationship, and (iii) additional decision-making tools (see Table 2). Themes were discussed between participants who provided personal thoughts, emotions, and explanations of their personal experiences with physicians during consultations.

**Table 2:** The main impact on themes and sub-themes on DAs and proposal of intervention

	Contents	Positive impacts of DAs	Negative impacts of DAs	Proposals of intervention
1. Typology and number of information	Various preferences about receiving information on lung cancer: - The patients need to acquire knowledge about cancer symptoms, clinical therapies, and possible related side effects - The patients’ preference to manage treatments’ side-effects only when present - Patients want to have information about the effectiveness of cancer treatments	- Having all the information, due to the rapid change in lung cancer patients’ disease state, allows a clearer knowledge about disease progression, treatments, and their effects on a physical and emotional level. It leads patients to react to clinical difficulties and not be caught unprepared. Patients have the perception of being aware about disease progression, preparing in advance for possible consequences - Patients who are not aware of future side-effects are more able to manage their emotional issues related to the fear of cancer treatments	- The awareness of oncological symptoms and cancer progression can generate concerns with a detrimental effect on daily activities. A permanent brooding on possible adverse consequences, increasing a permanent state of discomfort	It is therefore necessary to introduce personalized interventions based on preliminary investigations relating to one’s preferences in order to put patients in optimal conditions to cope with the cancer disease Paying greater attention on how to cope with oncological side-effects in everyday life is mandatory, perhaps taking advantage of the experience of patients who have already dealt with them
2. Patient-and-doctor relationship	The relevant role of the patient-doctor relationship during medical consultations. Patients highlighted: - Their need of having trust in physicians or being directly involved in treatment decisions and clinical management - The opportunity of having a unique representative doctor or different physicians during medical consultation	Patients who perceive to be lacking in oncological skills could be emotionally sustained and guided by a physician in the clinical management and treatment decisions On the contrary, other patients prefer to have a medical team that makes decisions about their cancer interventions. It increases their feelings of being reassured and satisfied	The request to patients to decide among different oncological treatments can generate patients’ frustrations and fear Moreover, the presence of too many physicians involved in a patient’s cancer journey can provoke a sense of uncertainties, damaging the doctor-patient relationship	The relationship between doctor and patient could be established on the basis of each one’s needs, providing support without requiring excessive efforts to those patients who perceive discomfort Furthermore, doctors have to address patients’ preferences and values to solve their doubts and sustain them effectively A multidisciplinary team could promote clinical assistance and insights into the patient’s journey; at the same time, a reference doctor who could establish a closer bond with the patient could be necessary
3. Additional decision-making tools	The possibility of receiving additional informative materials (e.g., audio-recorded video and booklets) can: - Encourage patients to share medical information with their family - Promote individuals’ awareness related to the lung cancer disease - Avoid the search of other additional decision tools that can increase uncertainty and doubts	Informative material could help patients in explaining illness and treatment characteristics to their family, without the fear of making mistakes and reducing the related emotional difficulties Moreover, additional informative tools can promote individual’s awareness and sustain patients in remembering all information needed, reducing risky behaviors, such as internet consultation	General informative material, outlining only specific cancer information, can create greater confusion and generate doubts that cannot be immediately solved by a specialist, increasing the patient’s distress Furthermore, relying on informative materials instead of an interaction with a healthcare professional can lead to the fear of receiving misinformation	Informative material could play a relevant role in outlining important cancer-related information; however, it needs to be integrated with direct interactions with healthcare professionals for active discussions on issues that patients can experience in daily life

### Theme One: Typology and Number of Information

The present theme explored participants’ preferences in receiving medical information. Patients provided controversial points of view regarding the number and type of information that they would have received from their physicians. In this regard, three sub-themes were evidenced: (1.1) patients’ knowledge currently, (1.2) awareness about cancer progression, and (1.3) the oncological treatments’ effectiveness.

The first sub-theme referred to participants’ preferences regarding receiving information about the harms and benefits of lung cancer treatments. Most of the sample reported that they received all information needed regarding lung cancer therapies and their related side effects, as follows: “It’s important to know possible consequences of our therapy. Physicians illustrated them, so if a side effect arises you are prepared to handle it. If you are prepared, symptoms do scare you” (ID5) and “When I went to be treated by an oncological therapy, I asked doctors for a lot of information about my treatments because I wanted to know everything now. Most of all, I didn’t want physicians to hide anything from me” (ID7). In line with this, patients reported having a clear knowledge of oncological treatments’ effects on a physical and emotional level. Specifically, a cancer lung patient underlined that: “Information was very clear, I understood sufficiently what was happening on my body and how I can feel during oncological treatment” (ID9) and “All cancer information has been clear and appropriate. It helped me to accept the proposal of treatments, reducing anxiety” (ID18). Only one patient reported that: “Physicians shared with me all information regarding illness and its treatments. However, there was sometimes conflicting information” (ID20). On the contrary, others evidenced that, despite all information being clear, they perceived a confusional mental state. Despite a participant presenting her preferences in knowing illness treatments and future consequences, she expressed difficulties in illness acceptance: “When the disease progresses, symptoms become very hard, and you need time to accept. However, I’d rather prefer to know than be unconscious” (ID11). In particular, illness acceptance was strongly linked to several difficulties in managing daily issues after cancer; this way, patients would have preferred to know how to cope with side-effects at the beginning of their oncological journey: “I would have preferred more information on how to handle side effects in daily life, for example regarding everyday activities that I could have continued to do or not (e.g., physical exercise and walking” (ID15). As mentioned by the previous participants, lung cancer patients stated that they would have also known other information, not only strictly linked to lung cancer treatments. In particular, they would have received indications about how to manage their everyday life (e.g., playing sports and physical activities, needing essential restorations, and a dietary regime), as follows: “I got all the information about medications, of course. However, I would have needed to receive information about the outline of my day, if I could keep doing what I did before illness or not., even the simplest things. For example, nobody told me if I could go walking or to the swimming-pool!” (ID2). Accordingly, another participant reported that, even if all oncological information were provided, he would have been more aware of how to manage oncological side-effects, such as tingling of the feet and vomiting, expressing high emotional distress: “It’s not so much the explanation about the treatment, also because we have no other alternatives. We must do it, and we have no choice to refuse or syndicate. Physicians also explain the possible side effects; however, I would have needed to clearly understand how to cope with side effects that make daily life tiring. It’s as if the possible side-effects are usual and taken for granted, for example the tingling of the feet, sense of vomiting, just because we are sick” (ID3). Contrarily, other participants said that they preferred not to have information about their cancer because they feel better now (“I don’t want to know anything, I really don’t want to know anything! I consider myself a healthy person, I haven’t had any side effects, so I’m fine” (ID1)) or due to the fear of being aware of oncological symptoms and consequences (“I also ask very little because unfortunately I know the disease very well, being a very present disease in my family history. I know how it can evolve and what could happen to me, how sick I could be, so I don’t want to receive other information” (ID11)). More specifically, a participant expressed her need to not think about oncological information and related emotions at home, involving efforts and time-dedicating to this topic only with her physician: “I ask as little as possible, I trust them because I believe in medicine. I try to follow what they tell me, but they still explain everything and ask me many questions. But when I go out of there I close. I go home as if I was never there. That’s my reaction” (ID12). In addition, having previous experience with lung cancer as a caregiver impacts the interest in receiving oncological information. In accordance with this, a patient stated she already knew all the information needed; thus, she did not want to receive other information, being satisfied by what she already knew: “The information was very clear, and I understood sufficiently what was going on and what I can expect. I am satisfied that I knew everything I needed to know” (ID4). Lastly, only one participant stated that she did not receive all the information clearly, even if she would like to know symptoms and their related medical consequences: “I didn’t have all the information about side effects; physicians didn’t refer them to me. I would ask but they would tell me it could happen as well as not, so you were left in uncertainty” (ID6).

The second sub-theme was related to patients’ desire to be aware of cancer progression and its related symptoms. Some patients reported that they preferred to be unknown about cancer progression and its related symptoms. Specifically, a woman highlighted that being aware of cancer progression characteristics impacted her emotions negatively, as follows: “When there is progression, it is heavy to hear about. I didn’t find it very positive to know everything, despite the physicians’ nice manners” (ID2). A woman highlighted the relevance of communicating the adverse effects of cancer treatment more appropriately and paying attention to not increase anxiety and fear in patients: “I would suggest that it would be important to specify what the adverse effects of treatments could lead, addressing the one-to-one point. Listening to all the adverse effects is a shock and scary!” (ID20). At the same time, other participants evidenced that knowing expected cancer progression symptoms would have been useless, considering the rapid change in their disease state: “I have experienced so many of those enthusiasms that then catapulted into the opposite within a short time, or so many situations that were dramatic and then resolved. So, I’ve realized that it’s a matter on which you must stay cautiously” (ID13). Accordingly, a man stated that he preferred to cope with symptoms step by step, without knowing what could have happened in advance. He explained to the group that it had been his emotional strategy to deal with fear and worry only when necessary due to cancer progression and the implementation of other oncological treatments. In other words, he preferred to know how to manage symptoms only when currently present, as stated in the following sentence: “Some people prefer to know everything beforehand and be clear in everything that may happen. I deal with them when they happen to me, rather than knowing everything before” (ID8). Similarly, another participant evidenced the risk of knowing future symptoms of cancer progression in advance due to the fear of living with anxiety and worries every day: “I did not want to know possible side effects because I would be standing there waiting for the moment when the pain came, the burning. Suppose I would have known possible side effects. In that case, I’ll be emotionally in trouble in advance” (ID1). Otherwise, there were some patients who preferred to know all information also regarding lung cancer progression and its related future consequences in terms of treatments and side-effects: “I have always preferred to know everything about my treatment, especially the possible side effects, my future prospects, and probability of treatment effectiveness, also regarding cancer progression” (ID5).

The third sub-theme is related to knowing the effectiveness of cancer treatments. Patients reported receiving all information about the efficacy of treatments, which is important to increase trust in positive outcomes. For example, participants said: “In my opinion, it has been important to receive information regarding the effectiveness of my cancer treatments. Understanding that it would be effective to stop cancer progression was fundamental to foster hope and my adherence to this treatment” (ID16). Accordingly, another participant reported the need to collect information about cancer treatments and their efficacy to be aware of what was happening and if would be the best choice possible, as follows: “I received a lot of information, and I think that they were appropriate and helpful for me to know my disease. However, I needed to receive further details about the effectiveness of my cancer treatment because I wanted to be sure that it would be the best option for my diagnosis” (ID17).

### Theme Two: Patient-and-Doctor Relationship

The second theme was related to the role of the patient-and-doctor relationship during medical consultations. All participants reported great satisfaction with cancer care received and the health care system. In particular, a patient said: “Oncologists and nurses of my hospital have better skills compared to other hospital staff; the team is very kind and attentive to meet all the patient’s needs” (ID3). According to this opinion, other patients said that “the team makes you feel at home, you forget to be in a hospital” (ID13) and “I can say that the oncologists of the institute are wonderful! They have a high level of professional skills, empathy, professionalism, and availability” (ID10). Starting from this overall background, two relevant sub-themes emerged as follows: (2.1) having trust in their physicians and (2.2) the need for a unique point of reference during medical consultations.

The first sub-theme of the second macro-category highlighted that many participants trusted physicians and their related decisions. Patients expressed their need to be guided by physicians in treatment decisions because of their lack of oncological skills. In particular, patients said that: “I trust the doctors, and not being from the field I can’t contradict them because I don’t have the expertise to do so” (ID4). Other patients thought that “the level of understanding depends as much on how much the patient asks about his or her treatment. I don’t ask so much. I trust what doctors tell me, not being an expert in this area” (ID1). This possibility promoted patients’ quietness and hope in positive outcomes, recognizing physicians’ decisions as the best possible for them, as follows: “I didn’t ask a lot of information because I relied, as I am wont to do. I rely, physicians will do what they think is best” (ID12). This is in line with the overall need to be emotionally sustained by physicians, as reported by participants. In this regard, a man appreciated that his physician asked him what his questions were, stimulating the discussion: “Every time I go, they ask me: ‘Today tell us what you want to ask us?’ In this way, they stimulate me to ask them questions, it’s an incredible thing” (ID7). Thus, the group evidence the relevance of having trust in the healthcare system and its related professionals who can make the best treatment decisions. A patient underlined: “From first oncological consultations, physicians conveyed us a pleasant confidence. They seemed very confident in handling the situation” (ID14). In line with this, several patients highlighted the importance of having a strict and personal relationship with their physicians: “My relationship with my physicians is fundamental to solving doubts. I need to discuss with my physicians what is happening because I don’t want to have doubts, which increase anxiety and distress” (ID17) and “I think that patient-and-doctor communication in presence is very productive. In my experience, meeting a group of oncologists who were prepared and available to discuss with me about cancer’s contents gave me hope and trust in positive outcomes of my cancer treatments” (ID18). On the contrary, lung cancer patients mentioned frustrations and fear when physicians asked them to decide among different oncological treatments. In this regard, a woman stated that she preferred to consult several physicians before deciding on her care, increasing frustrations and negative emotions. She explicitly said that she would have preferred that the first physician made decisions: “Sometime, they asked me to decide among two possible treatments…at that time, I really struggled with them, because I had to consult several doctors…this has annoyed me because I hoped the doctor would be the one to advise me and choose best solution for me” (ID2).

Regarding the second sub-theme in this macro-category, patients discuss for a long time during the focus group intervention regarding their preferences for having one or more physicians during the medical consultation process. Most of the sample experienced the presence of more than one physician to discuss their oncological treatment decisions and future steps. In this regard, lung cancer patients stated the relevance of the relationship with physicians can influence oncological information awareness. Specifically, they highlighted the importance of having trust in physicians, recognizing that a multidisciplinary équipe that discusses and makes decisions about their cancer interventions had reassured them. Patients underlined: “I felt comforted to see that final decisions were chosen by a multidisciplinary team and not by a single individual” (ID6). In particular, a woman mentioned that, despite her initial confusional state and cognitive disorientation, she appreciated that all the medical équipe knew her clinical situations and was able to respond to her doubts, without providing contradictory information, as follows: “Although there is a referring doctor, I know that the team communicates with each other…so whenever I ask for something, I notice that all physicians have information and follow up with you well, although they take turns with each other” (ID13). Similarly, another woman evidenced the benefits of having multiple points of view by different professionals: “In my option, to be followed by a multidisciplinary équipe is very advantageous because you have the opportunity to get information from different points of view…it has a global view of your disease, I appreciate this” (ID12). On the contrary, others stated that they preferred having a unique physician as a point of reference. They reported frustration and a sense of uncertainty due to the presence of too many physicians involved in their cancer journey: “I honestly would have preferred to have one referring doctor rather than the involvement of the whole équipe. At every checkup, I met different doctors” (ID3), “One always looks for a reference point to be reassured… it is always important to be reassured by the same doctor” (ID6), and “I even perceived the need to have a unique point of reference during my oncological treatments. I need to be reassured, although each physician I met clearly communicated with me. I would have preferred to have only one oncologist to be sure to be followed in my oncological treatment in the best way possible” (ID21). Specifically, a participant reported that meeting many physicians for consultations reduces the possibility of having a human and deep relationship: “I don’t like the fact that in each visit, I find a different doctor because you don’t establish that human relationship that can be established with a regular doctor” (ID4) and “I think they are great, all of them and they are all very nice, all of them, as the other ladies were saying, however, I would have liked more to have a referral doctor who didn’t change at every visit, with whom to have a more direct human relationship” (ID9).

### Theme Three: Additional Decision-Making Tools

The third theme involved patients’ preferences regarding the possibility of receiving additional decision-making tools (e.g., audio-recorded video and booklets) to make decisions on health treatments. Interestingly, patients reported conflicting opinions on this topic. Specifically, three sub-themes emerged, as follows: (3.1) sharing information with their family, (3.2) the promotion of individuals’ awareness, and (3.3) additional decision tools as emotional issues.

The first sub-theme in the third macro-category related to adding decision-making tools evidenced that sharing medical information with other family members is often perceived as a source of emotional difficulties. This way, some participants reported that having a brochure, for example, could help them in explaining illness and treatment characteristics to their significant others, as follows: “In my opinion, a booklet would have been very helpful, especially to share the information with their family members and explain to them treatments and how they work” (ID8). On an emotional level, lung cancer patients who meet physicians could perceive a low level of anxiety in capturing all information presented, as stated: “I always prefer to ask my doctor and clarify my doubts because a brochure would increase my fear and anxiety” (ID2). Accordingly, a brochure must make some patients less worried about communicating information to their relatives and family members, without the fear of making a mistake: “I would have been more comfortable to present medical information to my family using a brochure…I would have not the risk to misinterpret the information that I got from doctors or forget it” (ID1). Finally, another participant stated that a brochure would have been helpful in sharing information with family members and perceiving their emotional support; however, she highlighted the relevance of having more meetings with professionals to address negative emotions and learn how to manage everyday issues, as follows: “In my opinion, having something paper-based to consult and share with one’s family members would be a nice idea, although I argue that more meetings among ourselves with the support of experts should be conducted” (ID8) and “I would have preferred to receive a brochure with the contents related to my disease. It would have been helpful to understand better what was happening…during the medical consultation, my husband and I were too emotionally involved and shocked. It would be beneficial to have the contents of the first consultation at home to discuss each other on them” (ID20).

In line with it, the second sub-theme in this macro-category highlighted that additional decision tools would have promoted individuals’ awareness, reducing internet consultation. A patient mentioned that, when information communicated by physicians is too complicated and unintelligible, she usually searches for information on the Internet: “When I pick up the CT scan or Pet scan, I consult google to understand what it says…when it is easy to understand okay (…) but sometimes there are difficult words to understand, and I get a little alert” (ID5) and “I would have received decisional tools to consult at home only if it would contain concise and easy-to-be-read contents. On the contrary, decision tools would generate confusion and doubts” (ID18). However, others reported that searching medical information on the Internet could have been a risky behavior due to various reasons: for example, it would have generated a confusional state because they are not able to understand and interpret this information: “I used to consult the Internet. I did it for a long time, then I stopped because you don’t have the skills to understand all the way through anyway, so it confuses you” (ID7) and “After receiving a diagnosis, I searched on the Internet for new types of cancer treatments. However, I noticed that oncologists were the best people with whom to share my questions” (ID18). This way, additional decision tools would have been helpful to understand and remember all information needed, avoiding asking information to significant others or someone else (e.g., a lung cancer peer or another health care professional for a second opinion) and reducing anxiety and uncertainties. A patient declared: “In my opinion it would be a very good idea, so one doesn’t go looking for information, but having the brochure one can consult it more leisurely at home” (ID12). Furthermore, reading information at home and with a more balanced level of emotions would have led to access to oncological information being quieter and more aware: “For me, a brochure would have been helpful in resolving many doubts that arise immediately after the consultation, when you go home and think again about everything” (ID15). Lastly, a patient highlighted that: “Having information to read at home could help provide further details about managing our daily lives and the best practices to deal with the disease. I would not have received information regarding illness; I think that this type of information would have increased my anxiety” (ID18).

Finally, the third sub-theme related to the additional DAs’ macro-category evidenced that tools could improve emotional issues. A group of participants were opposed to the integration of additional tools. They generally preferred not to receive other information, in addition to those provided by physicians: “No, I think that it isn’t a good idea, I prefer to ask all the things, I don’t go looking on the Internet or other people for fear of giving me wrong information. I only ask doctors because they know how to deal with the situation” (ID7). One possible reason was the fear of receiving misinformation or not understanding some contents of the decision tools, generating anxiety and doubts; thus, they preferred to listen only to the physician’s description of the illness state and its related treatments: “I would not have received a tool with information on my cancer because possible unclear contents would have enhanced anxiety and distress without the possibility of discussing it with my physicians suddenly. Usually, I have preferred to receive all information during medical consultation to solve my doubts in person” (ID19). In line with the relevance of the patient-and-doctor relationship, some lung cancer patients preferred not to search for additional decision tools because they were satisfied by their physician’s response: “I don’t look for other support to look for information because I trust in my hospital” (ID2).

## Discussion

The present focus group study explored lung cancer patients’ preferences in receiving information and their overall needs during medical consultations. Psychologists with expertise in psycho-oncological issues conducted three online focus groups to collect data about patients’ experience of the shared decision-making process and their related emotions. The findings of the present study present positive and negative outcomes of the current medical consultation characteristics on DAs. This is in line with our study that aims to develop effective DAs for lung cancer patients. Overall, obtained results revealed three main themes and eight sub-themes, which emerged following a bottom-up approach.

The first theme focused on the typology and the number of oncological information that patients would have received. Most of the sample reported that they obtained all information about illness characteristics and treatments they needed to know. However, all focus groups evidenced controversial opinions regarding the patients’ request to be aware of current knowledge and information related to cancer progression: some participants reported their need to know all available information to be aware of their illness characteristics and future consequences. In particular, some of them stated that they preferred to know the side-effects of oncological treatments in everyday life. Similarly, some of them said that they would have received physicians’ instructions about how to cope with physical changes. This is in line with the scientific literature that highlights the relevance of patients’ information seeking behaviors to personalized cancer therapies [40]. Indeed, levels of patients’ knowledge impact their treatment choice and adherence to trial studies [41]. In this case, patients express that having all cancer-related information allows a clearer knowledge about disease progression, treatments, and their effects on a physical and emotional level. This way, patients can react to clinical difficulties and not be caught unprepared, managing daily issues. On the contrary, other participants mentioned that they would have preferred not to receive information about their illness characteristics and related effects in the long run. In this regard, having oncological information could have increased emotional disorders, especially anxiety and fear of cancer recurrence. For example, it can provoke a permanent brooding on possible adverse consequences, leading to a constant state of discomfort. Patients strongly affirmed that being too aware would have damaged their adherence to treatment because of high levels of worry and hopelessness. In this regard, literature showed the relevance of addressing emotional issues, such as anxiety and depression among cancer patients [42]. Eraslan et al. [43] affirmed that reducing a patient’s severe fears and uncertainties associated with cancer diagnosis and treatments is fundamental to coping with cancer and promoting a positive emotional well-being. Interestingly, participants who have had previous experience with lung cancer in their families (e.g., relatives or parents facing lung cancer and its related symptoms in the previous years) did not want to receive other information. They reported well-known lung cancer characteristics and effects, without the need to further explore this topic with physicians again. As known, caregivers follow the oncological journals of patients strictly [44]. Caregivers are not only aware of the illness process and future steps of treatments but also become an essential support to promote patients’ adherence to treatments and emotional well-being [45]. In line with our results, current research indeed evidenced the prevalence of caregivers’ negative emotions, in terms of anxiety and depression especially, confirming their strong involvement in patients’ knowledge and decisions [46]. Psychological interventions could introduce personalized programs focused on preliminary investigations to arise patients’ preferences and make the best condition possible to cope with the cancer disease. Particularly, experiences of lung cancer patients who had oncological treatments in the past could be strongly relevant to tailored current DAs.

Second, focus groups highlighted the importance of the relationship with physicians to be emotionally sustained by the health care system of reference. Since the overall satisfaction regarding the oncological care received, participants stated they needed to trust their doctors. The lack of medical competencies leads lung cancer patients to prefer that physicians make the last decision regarding their oncological treatments. At the same time, patients shared various opinions: on one side, some patients needed to be involved in their cancer journey, asking for better knowledge about their current and future symptoms. On the other side, others explicitly said not to be involved in a shared decision-making process due to the fear of being unable to make the best health choice, reporting anxiety and frustration. Accordingly, a study by Köther et al. [47] demonstrated that emotional distress systematically affects patients during medical consultations, predicting decisional conflict and reducing their active involvement in their process of care. Of note, Amundsen et al. [48] evidenced that physicians’ behaviors are not always associated with the active involvement of patients in their medical consultations. The authors indeed showed that, despite a low physician’s shared decision-making behavior, some patients were active by asking questions to solve their doubts. This is relevant to consider patients’ characteristics and personality traits too. Furthermore, patients who participated in focus groups discussed having a unique physician of reference rather than the overall équipe. Although all lung cancer patients highlighted their overall satisfaction with their health care providers, some participants stated that they preferred to be treated by a unique physician. In particular, they expressed the need to have a more personal and intimate relationship with the doctor. The presence of too many physicians involved in the patient’s cancer journey can provoke a sense of uncertainties, damaging the doctor-patient relationship. Literature underlined the relevant role of an improved doctor-patient relationship to promote a better QoL in cancer patients [49, 50]. Particularly, shared decision-making can sustain doctor-patient relationships by underscoring the need for increased patient involvement in their health decisions and improving QoL [51]. On the contrary, others reported that discussing with many physicians belonging to the same medical équipe strongly reassured them. Specifically, they appreciated the possibility of receiving multiple opinions and were reassured of being treated by a group of specialists compared to only one physician. In this regard, Kedia and colleagues [52] who interviewed lung cancer patients in focus groups highlighted the positive perception of multidisciplinary care, which was experienced as more effective, safe, efficient, and patient-centered than standard care. Furthermore, the authors evidenced that multidisciplinary care increased the perception of physicians’ interest in patients’ needs, reducing their stress, confusion, and anxiety. Psychological intervention must consider the relevance of the patient-doctor relationship based on each one’s needs. Particularly, clinicians could provide support without requiring excessive effort to those patients who are already in a condition of discomfort, for example. At the same time, addressing patients’ preferences and values could solve their doubts, promoting the perception of being sustained and satisfied. Lastly, patients’ preferences could evidence the need of involving a multidisciplinary team, which could promote clinical assistance and insights into the patient’s journey, or a reference doctor who could establish a closer bond with the patient.

Finally, the third topic addresses the possibility of receiving additional decision-making tools during medical consultations (e.g., a brochure, a booklet, or an audio-recorded video). Patients’ contrasting opinions emerged. Some people said that they would have needed a brochure as practical support to better explain illness characteristics and the subsequent oncological intervention to their families, without making mistakes. In this regard, patients shared their confusional mental state during medical consultation due to the strong impact of the oncological diagnosis on their emotions, in terms of anxiety and distress especially. This way, having additional practical materials could have helped them to discuss their oncological condition at home, without the fear of reporting wrong or inaccurate information. Of note, information sharing and communication within families generally cause several emotional issues because of cancer-related uncertainty and fear [53]. Authors evidenced that discussion about cancer can increase dilemmas, inhibiting cancer information exchange with family members. The implementation of devised strategies can be helpful in managing practical demands and avoiding the perception of a lack of requested information for example [54]. Interestingly, we must consider individual characteristics, such as people with greater self-efficacy are more likely to share cancer risk information and communicate more frequently with their relatives [55]. Similarly, an additional decision-making tool could have helped them to increase their personal awareness about their illness, avoiding searching for information on the internet platform. As reported by patients, a brochure and/or an audio-recorded video would have solved patients’ doubts and misinformation, especially when they did not understand some physician’s words during consultation. In other words, additional informative tools can promote patients’ awareness in remembering all the information needed, reducing risky behaviors such as internet consultation. In this regard, studies demonstrated that advanced educational materials and DAs for lung cancer patients can sustain them to understand the illness process, making the best choice of treatment [40, 56]. Moreover, additional tools can reduce patients’ confusional state and high anxiety levels. In particular, the implementation of tailored devices can reduce patients’ worry, promoting individual awareness of the illness process to get the most favorable outcomes possible [57]. On the contrary, other participants reported that they would not have to receive other additional decision tools because they were satisfied with the current information received. Interestingly, they were worried about the risk of becoming confused due to the involvement of other sources of information. In this regard, scientific literature demonstrated that too much information could confuse patients. It is paramount to deal with information presented by the DAs, which may be integrated with a medical consultation in which patients can experience a shared decision-making process with their physician [58]. Relying on informative materials instead of an interaction with a healthcare professional can lead to the fear of receiving misinformation. Psychological intervention could involve the implementation of DAs tools as informative materials to outline important cancer-related information; however, DAs tools need to be integrated in line with direct interactions with healthcare professionals to promote active patient-doctor discussions on cancer-related issues daily.

The present study is not free of limitations. First, it is not possible to rule out that the participants’ social conscience was not at least partly influenced by social desirability bias, which can often be encountered in focus group studies [59]. However, the literature showed that conducting focus groups online can reduce social desirability bias thanks to participants’ congruences (e.g., all participants provided personal disclosures) and the sharing of extensive details related to the purpose of the research by conductor(s) [60]. It is also worth noting that our recruitment involved only lung cancer patients from Istituto Nazionale dei Tumori (Milan), which limits the transferability of the study results. Future research could explore patients’ opinions and preferences who are affected by other cancer types as well as people who have been treated in different hospitals. Lastly, the present study does not provide specific characteristics of patients including income and oncological treatments, for instance.

## Conclusion

In conclusion, lung cancer patients who participated in focus groups presented various opinions regarding the implementation of DAs during medical consultation. Participants only sometimes wanted to know everything about their illness and oncological treatments, while others explicitly expressed the desire of being informed. Accordingly, the present contribution highlights the relevance of the patient-doctor relationship, in a one-to-one consultation as well as having the possibility of discussing with several physicians who belong to the same équipe. Overall, lung cancer patients reported their need to be guided during making health decisions, in a trusty and confident context. This is in line with the proposal of even more personalized medical consultations, which can address individuals’ characteristics and values. As a clinical implication, DAs would be helpful to promote oncological consultations prioritizing patients’ needs and preferences (e.g., supporting patients in communicating information with their families and being more aware of their illness information, if requested). At the same time, it is fundamental to assess lung cancer patients’ preferences in advance, avoiding the implementation of additional decision tools whether perceived as a source of distress and anxiety by patients. 

## Data Availability

Dataset are available under reasonable request.
